# Comparison of empagliflozin and vildagliptin for efficacy and safety in type 2 diabetes mellitus in the Pakistani population

**DOI:** 10.3389/fendo.2022.926633

**Published:** 2022-08-17

**Authors:** Asima Khan, Izhan A. Khan, Hussain Abidi, Mansoor Ahmed

**Affiliations:** ^1^ School of Public Health, Dow University of Health Sciences, Karachi, Pakistan; ^2^ Department of Medicine, Dow University of Health Sciences, Karachi, Pakistan; ^3^ Clinical Research Operations, Getz Pharma, Karachi, Pakistan

**Keywords:** type 2 diabetes mellitus, empagliflozin, vildagliptin, efficacy, safety

## Abstract

**Background:**

Diabetes mellitus (DM) is a chronic disease that needs early management to prevent complications and premature mortality. Therefore, it is essential to select evidence-based drugs available to control diabetes and limit the progression to related complications. This study aimed to compare the efficacy and safety of empagliflozin and vildagliptin in people with type 2 DM.

**Methods:**

This was an open-label, parallel randomized controlled trial (NCT 05359432) conducted at two tertiary care hospitals in Karachi, Pakistan. After obtaining consent, participants were randomized into two groups. The first group was given empagliflozin (10 mg once or two times daily) with metformin, and the second group got vildagliptin (50 mg once or two times daily) with metformin. HbA1c, high-density lipoprotein (HDL) levels, systolic blood pressure, fasting blood glucose, and body weight were measured at the baseline and 24-week visits.

**Results:**

A total of 120 patients fulfilled the selection criteria and then underwent randomization to be placed into empagliflozin and vildagliptin groups. The mean change in HbA1c (-0.97% ± 0.68 for empagliflozin and -0.82% ± 1.57 for vildagliptin) was statistically similar in both groups (p-value = 0.980). No statistically significant difference was observed between the two groups for safety parameters such as eGFR (p = 0.46), serum ALT (p = 0.13), LDL (p = 0.23), total cholesterol (p = 0.49), and triglycerides (p = 0.49).

**Conclusion:**

Results of the study highlight that vildagliptin and empagliflozin have a significant beneficial effect in reducing HbA1c, fasting blood glucose, systolic blood pressure, and weight of participants. Both drugs had no differences when compared on safety parameters.

## Introduction

Type 2 diabetes mellitus (T2DM) is a long-term disorder that substantially influences an individual’s wellbeing along with the quality of life and is associated with the increasing health burden worldwide (1). The World Health Organization states that in recent times DM has risen to become the most common cause of death globally, with a global mortality rate of around 1.5 million in 2019 (2). Furthermore, it is estimated that the prevalence of DM is 9.3% (463 million of the population) around the world and is expected to rise to 10.2% (578 million) in the year 2030 (1). The International Diabetes Federation (IDF) reported that over 19 million adults in Pakistan are estimated to have diabetes, but during the last 2 years, 13.6 million new people have been diagnosed, bringing the total to over 33 million. There are additional 11 million adults reported with the impaired glucose tolerance, and 8.9 million people are with undiagnosed diabetes which is exceptionally alarming for health professionals and policymakers (3). According to the 10th edition of the IDF Diabetes Atlas, Pakistan has now superseded the United States of America and is currently placed third in the world after China and India (4, 5).

Poorly controlled diabetes can affect a range of organ systems in the human body, leading to life-threatening complications over a course of time. These include microvascular (nephropathy, neuropathy, and retinopathy) as well as macrovascular complications (cardiovascular diseases and peripheral vascular diseases) (6, 7). The factors that increase the mortality risks in diabetic patients include cardiovascular disease, hypertension, increased serum cholesterol, and cigarette smoking (8). Many patients with T2DM remain undiagnosed for many years, and they may acquire diabetes-related complications or die even before diagnosis (9).

Essential components of effective management of diabetes include pharmacological interventions and lifestyle modification. To date, various types of oral and hypoglycemic parenteral medicines are available to attain control of glycemic levels in T2DM patients. However, in the majority of patients, the combination of several hypoglycemic drugs to achieve optimal glycemic control is required (10).

Several studies have been conducted to determine the safety and efficacy of oral hypoglycemic drugs (in addition to metformin) in T2DM patients. In the early 2000s in Pakistan, DPP-4 inhibitors and recently a newer class of drug, i.e., SGLT-2 inhibitor, have been introduced with cost-effective benefits for diabetes management. However, the available data are scarce to compare the safety and efficacy of DPP-4 inhibitors and SGLT-2 inhibitors especially among the Pakistani population. With the comprehensive management of T2DM, the extra burden of medications and associated complications is minimized while improving the quality of life. Different antidiabetic drugs are utilized for the correction of hyperglycemia and complications linked with T2DM. The efficacy and safety of SGLT2i and DPP4i drugs have been studied and compared globally. However, to the best of our understanding this comparison is not established in Pakistan yet. Such a study would rationalize evidence-based clinical judgment about the choice of safer and efficacious yet cost-effective oral hypoglycemic drugs. Therefore, the aim of this study was to compare the efficacy and safety of an SGLT-2 inhibitor, empagliflozin, and a DPP-4 inhibitor, vildagliptin.

## Methodology

This experimental, comparative, open-label, parallel, randomized controlled trial (NCT 05359432) was carried out at two different primary care centers in Karachi—Dr. Riasat Medical Centre Allah Wala Town and Sindh Government Hospital Liaquatabad. The trial commenced after obtaining approval from the institutional review board of Dow University of Health Sciences (IRB-1713/DUHS/Approval/2020/) on 21 November 2020 and ended on 01 November 2021. The entire study was conducted following the principles outlined in the International Council for Harmonization of Technical Requirements for Pharmaceuticals for Human Use – Good Clinical Practices (ICH-GCP, E6 R2) guidelines. The sample size was computed using the outcome (mean change in HbA1c %) in two study groups from a previously published study keeping a 95% confidence interval and 80% power (11). The sample size calculated for our study was N = 45 patients in each study group. Equating the dropout rate of 19% from the previous study, the adjusted sample size was N = 60 in each group. The sample size was calculated using an online calculator (12).

The inclusion criteria were any male or female patient diagnosed with T2DM of age 30–65 years with uncontrolled T2DM having >7% HbA1c levels. This age group was carefully selected as the prevalence of disease before the 30 years of age is lower and under 30; the preferred choice of therapy is different from the drugs compared in this study, whereas there is a higher risk of adverse events in people with diabetes above 65 years of age. Patients on monotherapy of metformin for the past 3 months on a fixed dose of 1,500 mg/day along with lifestyle modifications having a BMI range from 18 to 45 kg/m^2^ and eGFR ≥60 ml/min/1.73 m^2^ were included in this study. The exclusion criteria were pregnant or any woman planning to conceive in the coming 6 months, patients with type 1 diabetes or diabetes resulting from specific causes, and patients with advanced diabetic complications or any other terminal disease(s) requiring long-term medications. Moreover, patients involved in other trials on therapy with SGLT2 inhibitor or DPP4 inhibitor, patients on insulin or any other oral hypoglycemic drugs except metformin, and patients with eGFR levels ≤60 ml/min/1.73 m^2^ were excluded.

A simple random sampling technique was utilized to randomize the participants in the treatment group empagliflozin with metformin and vildagliptin with metformin. Patients were allocated to each study group in a 1:1 ratio using a computer-generated sequence for randomization. The study groups were divided into A (active comparator is empagliflozin and metformin with a dose of 10 mg once or twice daily) and B (active comparator is vildagliptin and metformin with a dose of 50 mg once or twice daily).

The data were gathered in an IRB-approved case report form (CRF) after getting written informed consent from patients. Patients were informed about the entire procedure as well as the potential adverse events and how to report them in local languages such as Sindhi or Urdu. The demographic data, weight, height, BMI, prior medical history or comorbidities, laboratory outcomes such as HbA1c, fasting blood sugar, lipid profile, and renal function parameters at baseline and the 24th week of treatment were recorded on CRF during the first and last visits. Blood samples were taken by a lab assistant for relevant laboratory tests.

Following the assessments, all patients were randomized into two groups, A or B. The proformas were color-coded with either yellow or blue for empagliflozin and vildagliptin, respectively. Lifestyle modifications and metformin continued. The study drugs were initiated with a low dose and intensified where needed as per routine clinical care to achieve better control. Participants or their caregivers were taught to check and write down blood sugar levels correctly. Patients were asked to return for a follow-up appointment at the end of each month (five follow-ups for a 24-week study period) to ensure treatment compliance. At the last follow-up, all the laboratory tests were repeated as were done in the baseline visit.

**Figure 1:**
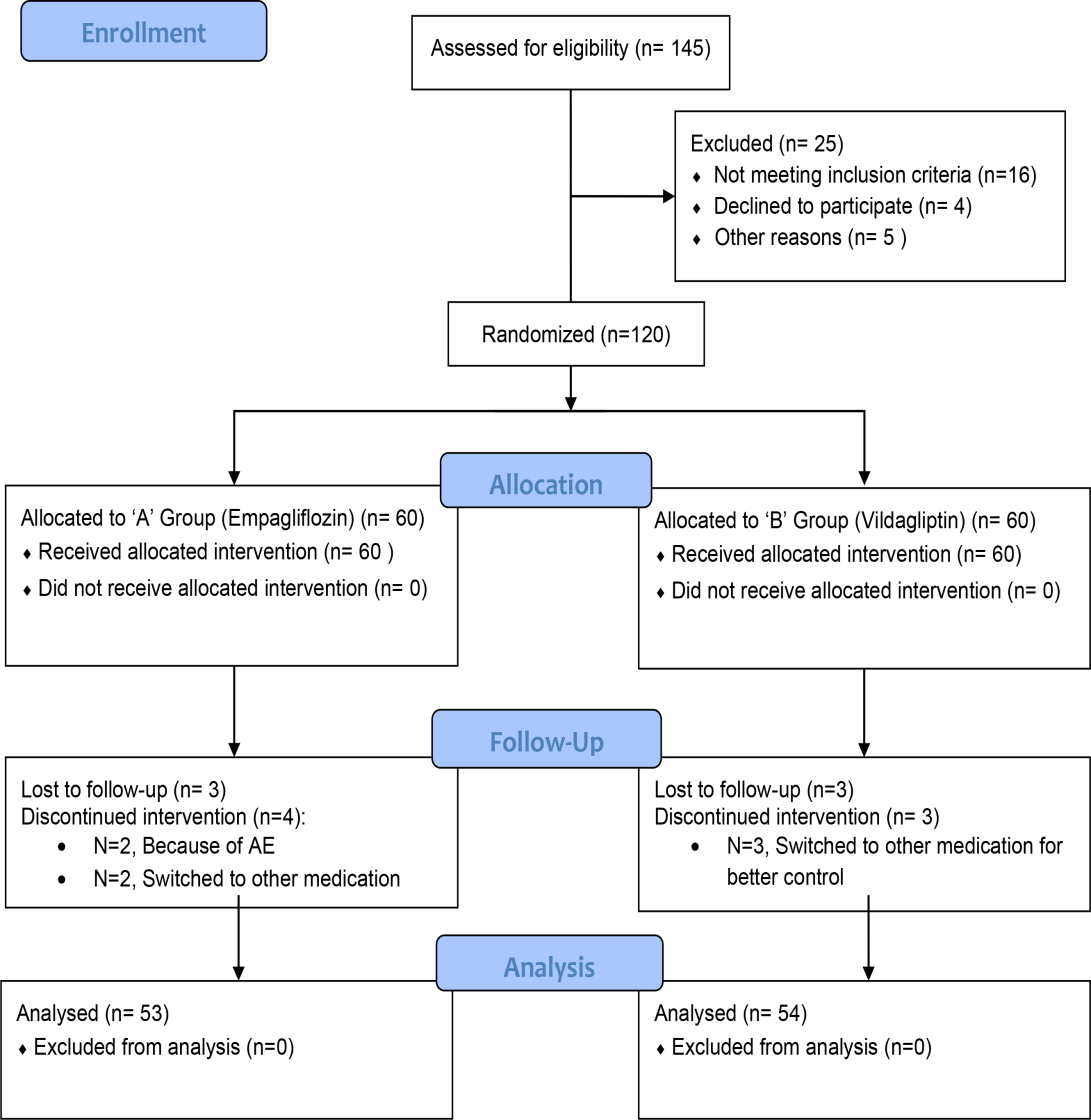
Consort flow diagram.

A daily patient diary was provided to keep a record of blood glucose monitoring at least three times a week. All patients were followed up *via* telephonic correspondence to keep track of any adverse events. **Figure 1** shows that the trial follows CONSORT (Consolidated Standards of Reporting Trials) guidelines to report the results and flow of the study.

Efficacy outcomes include the change from baseline to the 24th week of treatment in parameters in study groups A and B as a decrease in glycosylated hemoglobin A1c (HbA1c), systolic blood pressure, fasting blood sugar, and body weight. It also included an increase in HDL levels. The safety outcomes included reporting any adverse event or any serious adverse event during the trial. Furthermore, for adverse event reporting, a few critical renal, cardiac, and hepatic parameters were analyzed to ensure safety reporting, which included alterations in laboratory parameters from baseline to the 24th week of treatment. These included estimated glomerular filtration rate (eGFR), serum alanine transaminase (serum ALT), low-density lipoprotein cholesterol (LDL), total cholesterol, and serum triglycerides. Any adverse event was duly reported.

After completion of the study with baseline and 24-week readings, the data were entered and analyzed on IBM SPSS for Windows, version 21.0 (IBM Corp., Armonk, NY). The continuous variables were reported as mean and standard deviations, categorical variables as frequencies and percentages. Statistical significance was kept at α = 0.05.

The Kolmogorov–Smirnov statistical test was utilized to gauge the normality of the data. In this study, there was a non-normal distribution of data, which indicated the use of non-parametric tests for the determination of statistical significance. The Mann–Whitney U test was used to gauge the statistical significance for the comparison of continuous variables between the empagliflozin and vildagliptin treatment groups, whereas the Fisher’s exact test was used to gauge the statistical significance of the comparison of categorical variables. The Wilcoxon signed-rank test was used to compare the change for paired variables.

## Results

In total, 145 patients were screened. Out of that, 120 were eligible and were randomized into two study groups. However, because of the loss to follow-up and withdrawal, 107 patients completed the study. The mean age of the study participants was 52.28 ± 7.55 years and 49.4 ± 9.48 years in the empagliflozin and vildagliptin groups, respectively, with no statistical difference (p-value = 0.102) between the two. Gender distribution was equal (p-value = 0.268) in both study groups, with women accounting for 58.5% of the study population. Smoking, family history of diabetes, and comorbid conditions were also equally distributed in both study groups (p-value > 0.05). Baseline variables were comparably distributed in both study groups with no statistically significant difference (p-value > 0.05). Demographics of the study population are presented in **Table 1**.

**Table 1:** Demographics of the study population.

Participants’ characteristics		Mean ( ± S. D)		P-value*
		Empagliflozin	Vildagliptin	
**Age (years)**		52.28 ± 7.55	49.4 ± 9.48	0.102
**HbA1c (%)**		9.47 ± 1.44	9.18 ± 1.30	0.344
**eGFR (**ml/min/1.73 m^2^ **)**		84.68 ± 29.54	87.31 ± 24.54	0.737
		**Number (%)**		P-value**
**Gender**	**Male**	28 (23.7)	21 (17.8)	0.268
	**Female**	32 (27.1)	37 (31.4)	
**Comorbidity**	**Hypertension**	33 (28.2)	30 (25.6)	0.580
	**Cardiovascular disease**	1 (0.8)	0 (0)	1.000
	**Arthritis**	2 (1.7)	0 (0)	0.496
**Smoking**		16 (13.7)	8 (6.8)	0.108
**Family history of diabetes**		36 (34)	35 (33)	1.000
**Marital status**	**Married**	43 (37.7)	52 (45.6)	0.329
	**Unmarried, separated**	11 (9.6)	08 (7.0)	

*Mann–Whitney U test was used to gauge the statistical significance using α = 0.05. **Fisher’s exact test was used to gauge the statistical significance using α = 0.05.

Empagliflozin showed a reduction of 0.97% ± 0.68 in HbA1c (p < 0.001), and vildagliptin showed a reduction of 0.82% ± 1.57 in HbA1c (p < 0.001). The change in HbA1c was statistically comparable in both study groups. The HDL concentration was increased in both groups (0.90 ± 0.91 in the empagliflozin group vs. 2.70 ± 3.84 in the vildagliptin group). However, there was no statistically significant difference in the HDL concentration between the two groups from baseline to week 24.

Change in systolic blood pressure (- 13.14 ± 13.29 mmHg for empagliflozin and - 10.75 ± 12.22 mmHg for vildagliptin), change in fasting blood sugar (- 43.90 ± 35.5 mg/dl for empagliflozin and - 48.90 ± 41.98 mmHg for vildagliptin), and change in body weight (- 1.15 ± 2.56 kg for empagliflozin and - 1.03 ± 4.24 kg for vildagliptin) are comparable in both study groups. The calculated p-values are given in **Table 2**.

**Table 2:** Change in efficacy and safety variables from baseline to week 24.

Variable	Mean ± S. D		P value*
	Empagliflozin (N = 53)	Vildagliptin (N = 54)	
**Change in HbA1c (%)**	- 0.97 ± 0.68	- 0.82 ± 1.57	0.980
**Change in HDL**	0.90 ± 0.91	2.70 ± 3.84	0.303
**Change in systolic blood pressure (mmHg)**	- 13.14 ± 13.29	- 10.75 ± 12.22	0.396
**Change in fasting blood sugar (mg/dL)**	- 43.90 ± 35.5	- 48.90 ± 41.98	0.635
**Change in body weight (kg)**	- 1.15 ± 2.56	- 1.03 ± 4.24	0.517
**e GFR (**ml/min/1.73 m^2^ **)**	90.36 ± 29.88	94.67 ± 29.51	0. 46
**Serum ALT**	- 9.86 ± 21.66	- 3.56 ± 20.10	0.133
**LDL cholesterol**	- 15.44 ± 16.58	- 27.25 ± 22.90	0.235
**Total cholesterol**	- 27.38 ± 41.47	- 28.03 ± 50.92	0.497
**Triglycerides**	- 33.73 ± 54.94	- 37.96 ± 56.39	0.443

*Mann–Whitney U test to gauge the statistical significance for difference in two study groups using α = 0.05. - ve sign indicates the reduction.

The mean change in HbA1c (%) from the baseline visit to the 24th-week visit was statistically significant in both treatment groups. Empagliflozin showed a reduction of 0.97% ± 0.68 in HbA1c (p < 0.001), and vildagliptin showed a decrease of 0.82% ± 1.57 in HbA1c (p < 0.001), reported in **Table 3**. Empagliflozin showed a significant reduction of HbA1c (p < 0.001), from 9.42% ± 1.48 at baseline to 8.44% ± 1.08 at the 24th week, whereas vildagliptin also showed a reduction of HbA1c (p < 0.001), from 9.17% ± 1.36 at baseline to 8.35% ± 1.10 at the 24th week, as reported in **Table 3**. An increase in HDL concentration was found in both groups, and there was no statistically significant difference between the two groups in HDL concentration from baseline to week 24 (**Table 2**).

**Table 3:** Change in efficacy and safety variables from baseline to week 24.

Variable	Empagliflozin			Vildagliptin		
	Week 0	Week 24	P-value*	Week 0	Week 24	P-value*
**HbA1c (%)**	9.42 ± 1.48	8.44 ± 1.08	< 0.001	9.17 ± 1.36	8.35 ± 1.10	< 0.001
**HDL (mg/dL)**	44.8 ± 19.7	45.7 ± 6.5	< 0.001	40.9 ± 9.3	43.6 ± 8.7	< 0.001
**Systolic Blood Pressure (mmHg)**	135 ± 18.6	121 ± 12.8	< 0.001	131 ± 17.3	119 ± 10.6	< 0.001
**Fasting blood sugar (mmol/mL)**	166 ± 48	117 ± 26	< 0.001	175 ± 60	126 ± 31	< 0.001
**Body weight (kg)**	72 ± 11	70 ± 10	< 0.001	69 ± 10	68 ± 10	< 0.001
**eGFR (**ml/min/1.73 m^2^ **)**	84.68 ± 29.54	90.36 ± 29.88	< 0.001	87.31 ± 24.54	94.67 ± 29.51	< 0.014
**Serum ALT (IU/L)**	37.3 ± 18.6	27 ± 8	< 0.001	33.2 ± 15.7	29 ± 13	< 0.001
**Total cholesterol (mg/dL)**	225 ± 182	175 ± 56	< 0.001	212 ± 83	190 ± 53	< 0.001
**LDL (mg/dL)**	138 ± 43.6	123 ± 34.0	< 0.001	154 ± 34.8	127 ± 27.8	< 0.001
**Triglycerides (mg/dL)**	207 ± 119	169 ± 77	< 0.001	200 ± 66	161 ± 37	< 0.001

*Wilcoxon-signed rank test to gauge statistical significance for difference in paired sample α = 0.05.

The mean change within treatment groups for HDL (mg/dL) from the baseline visit to the 24th-week visit was statistically significant in both groups. Empagliflozin showed a significant increase of HDL (p < 0.001), from 44.8 ± 19.7 (mg/dL) at baseline to 45.7 ± 6.5 (mg/dL) at the 24th week, whereas vildagliptin showed an increase in HDL concentration (p < 0.001), from 40.9 ± 9.3 (mg/dL) at baseline to 43.6 ± 8.7 (mg/dL) at the 24th week, as reported in **Table 3**. The mean change within treatment groups for systolic blood pressure (mmHg) from the baseline to 24th-week visit was statistically significant in both study groups. Empagliflozin showed a significant reduction in systolic blood pressure (p < 0.001), from 135 ± 18.6 (mmHg) at baseline to 121 ± 12.8 (mmHg) at the 24th week, whereas vildagliptin also showed a reduction in systolic blood pressure (p < 0.001), from 131 ± 17.3 (mmHg) at baseline to 119 ± 10.6 (mmHg) at the 24th week, as reported in **Table 3**. The mean change for fasting blood sugar (mmol/mL) from the baseline to 24th-week visit within both treatment groups was statistically significant. Empagliflozin showed a significant reduction in fasting blood sugar (p < 0.001), from 166 ± 48 (mmol/mL) at baseline to 117 ± 26 (mmol/mL) at the 24th week, whereas vildagliptin also showed a reduction in fasting blood sugar (p < 0.001), from 175 ± 60 (mmol/mL) at baseline to 126 ± 31 (mmol/mL) in the 24th week, as reported in **Table 3**. The mean change within treatment groups for body weight (kg) from the baseline to the 24th week was statistically significant in both. Empagliflozin showed a significant reduction in body weight (p < 0.001), from 72 ± 11 (kg) at baseline to 70 ± 10 (kg) at the 24th week, whereas vildagliptin also showed a reduction in body weight (p < 0.001), from 69 ± 10 (kg) at baseline to 68 ± 10 (kg) at the 24th week, as reported in **Table 2**.

Safety outcome assessment first included reporting any adverse event during the study period. In this study, six (06) patients reported adverse events, all in the empagliflozin group. All adverse events were non-serious. Details of reported adverse events are illustrated in the **Supplemental Data**. The mean change in eGFR was statistically comparable (p = 0.465) in both study groups (90.36 ± 29.88 for empagliflozin and 94.67 ± 29.51 for vildagliptin). However, it was increased in the empagliflozin and vildagliptin groups after 24 weeks of therapy (p = 0.001) and (p = 0.014), respectively. The mean change in serum alanine transaminase (serum ALT) was greater in the empagliflozin group (- 9.86 ± 21.66) than in the vildagliptin group (- 3.56 ± 20.10) but with no statistically significant difference (p = 0.13) between the two (**Table 2**).

Among lipid profiles, a greater mean reduction in LDL concentrations was observed in the vildagliptin group (- 27.25 ± 22.90) than in the empagliflozin group (-15.44 ± 16.58). However, no statistically significant (p = 0.23) difference was present between the two groups. Similarly, the mean reduction in total cholesterol (- 27.38 ± 41.47 for empagliflozin and - 28.03 ± 50.92 for vildagliptin) and triglycerides (- 33.73 ± 54.94 for empagliflozin and - 37.96 ± 56.39 for vildagliptin) was also comparable in both groups with p-values of 0.49 and 0.44 for LDL cholesterol and triglycerides, respectively. Changes in safety outcome variables in both study groups are presented in **Table 2**.

The mean change within treatment groups for eGFR (mL/min/1.73 m^2^) from the baseline to 24th-week visit was statistically significant in both. Empagliflozin showed a significant increase in eGFR (p < 0.001), from 84.68 ± 29.54 (ml/min/1.73 m^2^) at baseline to 90.36 ± 29.88 (ml/min/1.73 m^2^) at the 24th week, whereas vildagliptin also showed an increase in eGFR (p < 0.004), from 87.31 ± 24.54 (ml/min/1.73 m^2^) at baseline to 94.67 ± 29.51 (ml/min/1.73 m^2^) at the 24th week, as reported in **Table 3**. The mean change within treatment groups for serum ALT (IU/L) from the baseline to 24th-week visit was statistically significant in both groups. Empagliflozin showed a significant reduction in serum ALT (p < 0.001), from 37.3 ± 18.6 (IU/L) at baseline to 27 ± 8 (IU/L) at the 24th week, whereas vildagliptin also showed a reduction in serum ALT (p < 0.001), from 33.2 ± 15.7 (IU/L) at baseline to 29 ± 13 (IU/L) at the 24th week, as reported in **Table 3**.

The mean change within treatment groups for total cholesterol levels (mg/dL) from the baseline to 24th-week visit was statistically significant in both study groups. Empagliflozin showed a significant reduction in total cholesterol levels (p < 0.001), from 225 ± 182 (mg/dL) at the baseline to 175 ± 56 (mg/dL) at the 24th week, whereas vildagliptin also showed a reduction in total cholesterol levels (p < 0.001), from 212 ± 83 (mg/dL) at the baseline to 190 ± 53 (mg/dL) at the 24th week, as reported in **Table 3**.

The mean change within treatment groups for LDL (mg/dL) from the baseline to 24th-week visit was statistically significant in both study groups. Empagliflozin showed a significant reduction in LDL (p < 0.001), from 138 ± 43.6 (mg/dL) at the baseline to 123 ± 34.0 (mg/dL) at the 24th week, whereas vildagliptin also showed a reduction in LDL (p < 0.001), from 154 ± 34.8 (mg/dL) at the baseline to 127 ± 27.8 (mg/dL) at the 24th week, as reported in **Table 3**. The mean change within treatment groups for triglycerides (mg/dL) from the baseline to 24th-week visit was statistically significant in both groups. Empagliflozin showed a significant reduction in triglycerides (p < 0.001), from 207 ± 119 (mg/dL) at the baseline to 169 ± 77 (mg/dL) at the 24th week, whereas vildagliptin also showed a reduction in triglycerides (p < 0.001), from 200 ± 66 (mg/dL) at baseline to 161 ± 37 (mg/dL) at the 24th week, as reported in **Table 3**.

## Discussion

For T2DM, the first line of management approach is lifestyle modification along with metformin (13). For uncontrolled diabetes mellitus patients, SGLT-2 inhibitors, DPP-4 inhibitors, and GLP-1 receptor agonists are regarded as first-line add-on medications to maintain glycemic control. Among three add-on antidiabetic medications, SGLT-2 inhibitors and DPP-4 inhibitors are significantly cost-effective options in comparison to GLP-1 receptor agonists. The two studied treatment options are well-researched worldwide (14, 15). Plenty of literature is available on the efficacy and safety of management of T2DM individually, but a head-to-head comparison of vildagliptin and empagliflozin is quite scarce. To the best of our knowledge, this is the first attempt to compare the two specified treatment options for the management of T2DM in Pakistan.

As per analyzed clinical data, both vildagliptin and empagliflozin significantly improved efficacy outcomes in study patients. Specifically, both drugs improved HbA1c and fasting blood sugar variables after 24 weeks of therapy. Statistical analysis also depicted that the improvement of efficacy parameters in both study groups is comparable, as no significant statistical difference was observed between the two groups. Our findings are in line with the studies conducted on vildagliptin and empagliflozin in comparison with other classes of antidiabetic drugs. In a 52-week multicentered, randomized, active-controlled trial, vildagliptin showed a significant reduction in HbA1c levels (16). In another study, vildagliptin showed better glucose lowering during a day in T2DM patients (17). Furthermore, in a 12-week dose-ranging, placebo-controlled, double-blinded trial conducted in participants with poorly controlled T2DM, empagliflozin showed promising results in the reduction of FBS levels and HbA1c levels (18). Empagliflozin also demonstrated similar results in a phase IIb study; a clinically substantial decrease in FBS and HbA1c was documented in the study participants (19). Thus, glycemic control with use of vildagliptin and empagliflozin along metformin are comparable in the management of T2DM.

Apart from glycemic variables, both vildagliptin and empagliflozin were also significantly associated with improvement in cardiovascular outcomes as well as HDL, which is an important biomarker for the prognosis of atherosclerotic cardiovascular disease. The study depicted that both vildagliptin and empagliflozin significantly increased HDL concentrations after the 24-week treatment. The decrease in systolic blood pressure was more remarkable in the empagliflozin group, but this difference is not statistically significant between the two, indicating that both treatment options have profoundly affected the reduction in systolic blood pressure as well. Our findings regarding the reduction in HDL with the usage of vildagliptin are comparable with the study conducted in Brazil in which patients who were given vildagliptin showed a higher HDL level at the end of the study (20). Another 24-week, randomized double-blinded, study was organized at 145 centers in eight different countries. These included the United States, four countries representing Europe, and three from Asia. The results showed a rise in HDL levels in patients who received vildagliptin (21). Consistent findings were observed in a randomized, active-controlled, open-label trial in which empagliflozin significantly increased the serum HDL level in patients with T2DM (22). Thus, both molecules improve HDL levels when given with metformin for 24 weeks or more.

The decrease in systolic blood pressure was more remarkable in the empagliflozin study group, but this difference was not statistically significant between empagliflozin and vildagliptin, indicating that both treatment options have reduced the systolic blood pressure. In a *post-hoc* data analysis from a phase III trial (n = 823) in T2DM and hypertensive patients receiving empagliflozin for 12 weeks and four phase III trials in T2DM patients (n = 2,477) receiving empagliflozin for 24 weeks, a significant reduction in systolic and diastolic blood pressures was reported (p-value < 0.001) (23). Moreover, data analysis of all reported double-blind, randomized controlled trials with vildagliptin monotherapy on drug-naïve T2DM patients receiving vildagliptin 50 mg once or twice daily (n = 2,108) showed a significant reduction in blood pressures (p < 0.0001) (24). Therefore, our results are consistent with findings from earlier studies.

Further, body weight is also an important measure for a better diabetes management, and reduction in body weight is now considered an essential parameter for recently introduced antidiabetic medications. Vildagliptin and empagliflozin also showed a clinically significant decrease in body weight. However, there was no statistically significant difference between the two groups. Our findings regarding the effect of empagliflozin on weight are consistent with several studies in which empagliflozin has shown a significant reduction in weight parameter (18). In contrast to our findings, several studies have shown treatment with vildagliptin being associated with no change in weight or associated with a small increase in weight (25–27). Thus, both drugs have proven effect in improving the body mass index (BMI).

Safety endpoints were also assessed during the study for both vildagliptin and empagliflozin, and both study groups were found to have similar safety profiles. In addition, only six minor adverse events were observed in the empagliflozin group. Out of six, only three were related to the study drug (perineal candidiasis, urinary incontinence, excessive urination, lethargy, nocturia) and the drug was discontinued in two patients due to adverse events.

To evaluate the renal safety of two drug options, eGFR changes were observed after a 24-week treatment period. Both groups showed an increase after the 24-week treatment. Therefore, this parameter is eventually depicting a comparable renal safety profile of two drugs for the management of T2DM. In the EMPA-REG OUTCOME trial (Empagliflozin Cardiovascular Outcome Event Trial in T2DM patients) assessing the effect of empagliflozin on clinical outcomes in T2DM patients with established cardiovascular disease and chronic kidney disease (CKD) and in 2,250 of 7,020 enrolled patients having prevalent CKD (with eGFR <60ml/min), addition of empagliflozin improved clinical outcomes and lowered mortalities (28). In the TREND study, which was a cross-sectional, non-interventional study on 1,918 Romanian T2DM patients treated with vildagliptin by 106 diabetologists from 36 countries, safe use in renal patients was considered extremely important (59.1%) after glycemic control, cardio-protection, and hypoglycemia avoidance (29).

The serum ALT biomarker was assessed to determine hepatic safety. The results showed that empagliflozin provided a better reduction in serum ALT levels than vildagliptin from the baseline visit to the 24-week follow-up with statistically significant outcomes. Thus, again the results represent a comparable hepatic safety profile of vildagliptin and empagliflozin in the Pakistani population. Several studies have also shown that the use of both empagliflozin and vildagliptin exhibited beneficial effects on the level of liver enzymes (30, 31). Lipid biomarkers are essential in gauging the prognosis of cardiovascular safety of diabetes mellitus patients. Cardiovascular safety is usually estimated in longitudinal clinical studies. However, this study determined lipid biomarkers before and after initiation of study treatment to assess cardiovascular safety. Based on the analysis, there was a significant reduction in total cholesterol, LDL, and triglycerides in 24 weeks of treatment in both groups. Among lipid biomarkers, vildagliptin showed a greater reduction in LDL, total cholesterol, and triglycerides than empagliflozin, but the difference between the two study groups is not statistically significant. Our findings are in line with several studies in which cardioprotective effects of vildagliptin and empagliflozin have been demonstrated by reducing serum total cholesterol, LDL, and triglycerides (32).

Based on the safety analysis, it can be maintained that both vildagliptin and empagliflozin have a similar safety profile in managing T2DM. Although more assessment parameters would gauge the safety profile even better, this study would eventually lead us to conduct more studies to address it.

### Limitations and strengths

This study has a few limitations. In our study, participants were required to keep track of their blood sugar levels on their own and record them in a diary. As many of them were not familiar with self-monitoring, they had to learn it. There was no means to validate whether they had documented the serum sugar levels correctly, and we had to depend on their learning skills. In addition to that, the literacy required to fill out the diary record amenably was one of the limitations during recruitment. Another constraint we faced was the need for participants to provide contact numbers for follow-up calls and visits. Many people regarded it as bothersome initially but later settled with an assurance of keeping complete privacy of cell phone numbers and minimum reminders. Moreover, due to intra-city travel bans and restrictions due to the COVID-19 pandemic, participants faced problems reaching sites at appointment dates. The duration of our study was 24 weeks, and T2DM is a chronic disease. Therefore, to determine the long-term effects of these drugs, more trials with longer durations in primary care settings are needed.

Our study has several strengths. One of which is that the comprehensive method applied in our study allowed us to prevent bias related to confounding factors and selection bias by randomization. In addition, the prospective design of the study reduced the recall error. Furthermore, our study allowed us to recruit patients from two different districts of the city. Moreover, to the best of our knowledge, this is the first study in Pakistan that compares the efficacy and safety of empagliflozin with vildagliptin in the Pakistani population. Our findings may have a substantial impact on the existing literature and areas of future research.

## Conclusion

When combined with metformin, the DPP-4 inhibitor (vildagliptin) and the SGLT2 inhibitor (empagliflozin) have a significant beneficial effect on diabetes management and play a vital role in lowering body weight and maintaining the lipid profile. They have also shown improvement in eGFR after 24 weeks. Management with vildagliptin and empagliflozin was tolerated well with a very low incidence of adverse events (AEs) and no incidence of serious adverse events. Furthermore, their beneficial effect on the lipid profile contributes to the prevention of cardiovascular disease in individuals with T2DM. Thus, either vildagliptin or empagliflozin presents an efficacious addition to first-line metformin for the effective management of T2DM in Pakistan.

## Data availability statement

The raw data supporting the conclusions of this article will be made available by the authors, without undue reservation.

## Ethics statement

The study involved human participants and was reviewed and approved by Institutional Review Board of Dow University of Health Sciences (IRB-1713/DUHS/Approval/2020/). The patients/participants provided their written informed consent to participate in this study.

## Author contributions

AK, MA, and IK contributed to the conception and design of the study. AK and IK participated in the acquisition of data, patient recruitments, consent, and organization of the database. HA performed the statistical analysis. AK and HA drafted the manuscript. MA critically reviewed the manuscript and contributed intellectual content. All authors read and approved the final version of the manuscript.

## Funding

The trial was sponsored by the Primary Care Diabetes Association, Pakistan. However, the institute had no role in the design of this research and collection, analysis, and interpretation of data, and writing of the manuscript.

## Acknowledgments

The authors are grateful to the individuals who took part in this study.

## Conflict of interest

Author HA was employed by the company Getz Pharma.

The remaining authors declare that the research was conducted in the absence of any commercial or financial relationships that could be construed as a potential conflict of interest.

## Publisher’s note

All claims expressed in this article are solely those of the authors and do not necessarily represent those of their affiliated organizations, or those of the publisher, the editors and the reviewers. Any product that may be evaluated in this article, or claim that may be made by its manufacturer, is not guaranteed or endorsed by the publisher.
