# Supplementary material for: Comparison of empagliflozin and vildagliptin for efficacy and safety in type 2 diabetes mellitus in the Pakistani population
Source: Front Endocrinol (Lausanne). 2022 Aug 17;13:926633. doi: 10.3389/fendo.2022.926633 (PMC9428695; doi:10.3389/fendo.2022.926633)
Supplement: Supplementary file 1 [file Table_1.docx]

| **Adverse events reported in Six (N=6) Empagliflozin group patients** | | | |
| --- | --- | --- | --- |
| **S. No** | **Adverse Event** | **Serious Adverse Event** | **Related to Study Drug** |
| 1 | Urinary Incontinence | No | Yes |
| 2 | Epigastric Pain | No | No |
| 3 | Excessive urination, lethargy, nocturia | No | Yes |
| 4 | Weakness, lethargy | No | No |
| 5 | Bloating | No | No |
| 6 | Perineal candidiasis | No | Yes |
